# Influence of humic acid and dihydroxy benzoic acid on the agglomeration, adsorption, sedimentation and dissolution of copper, manganese, aluminum and silica nanoparticles – A tentative exposure scenario

**DOI:** 10.1371/journal.pone.0192553

**Published:** 2018-02-08

**Authors:** Sulena Pradhan, Jonas Hedberg, Jörgen Rosenqvist, Caroline M. Jonsson, Susanna Wold, Eva Blomberg, Inger Odnevall Wallinder

**Affiliations:** 1 KTH Royal Institute of Technology, Department of Chemistry, Division of Surface and Corrosion Science, Stockholm, Sweden; 2 University of Gothenburg, Department of Chemistry and Molecular Biology, Gothenburg, Sweden; 3 KTH Royal Institute of Technology, Department of Chemistry, Division of Applied Physical Chemistry, Stockholm, Sweden; 4 RISE Research Institutes of Sweden, Division Bioscience and Materials, Stockholm, Sweden; Institute of Materials Science, GERMANY

## Abstract

This work focuses on kinetic aspects of stability, mobility, and dissolution of bare Cu, Al and Mn, and SiO_2_ NPs in synthetic freshwater (FW) with and without the presence of natural organic matter (NOM). This includes elucidation of particle and surface interactions, metal dissolution kinetics, and speciation predictions of released metals in solution. Dihydroxy benzoic acid (DHBA) and humic acid adsorbed rapidly on all metal NPs (<1 min) via multiple surface coordinations, followed in general by rapid agglomeration and concomitant sedimentation for a large fraction of the particles. In contrast, NOM did not induce agglomeration of the SiO_2_ NPs during the test duration (21 days). DHBA in concentrations of 0.1 and 1 mM was unable to stabilize the metal NPs for time periods longer than 6 h, whereas humic acid, at certain concentrations (20 mg/L) was more efficient (>24 h). The presence of NOM increased the amount of released metals into solution, in particular for Al and Cu, whereas the effect for Mn was minor. At least 10% of the particle mass was dissolved within 24 h and remained in solution for the metal NPs in the presence of NOM. Speciation modeling revealed that released Al and Cu predominantly formed complexes with NOM, whereas less complexation was seen for Mn. The results imply that potentially dispersed NPs of Cu, Al and Mn readily dissolve or sediment close to the source in freshwater of low salinity, whereas SiO_2_ NPs are more stable and therefore more mobile in solution.

## Introduction

The use of nanoparticles (NPs) in different applications is rapidly increasing due to their unique physical properties [1, 2]. This has resulted in increased production and widespread commercialization of nano-based products [3]. However, their small size also triggers a concern of potential adverse effects on the environment and on humans [4–6]. NPs of different kind and their released species can be dispersed to the environment in various ways, *e*.*g*. via disposal from various industrial processes, from consumer products, or via wear particles at traffic situations [7]. The dispersion of metal NPs has however so far not been seen to result in very high environmental concentrations [8]. The concentrations of most metal and metal oxide NPs are estimated to be less than 1 μg/L at aquatic settings, with *e*.*g*. TiO_2_ being a notable exception [8]. The increasing environmental concentrations [9] however underline the need for studies of the environmental fate of metal NPs dispersed from different applications in the society. These investigations should among other aspects assess an improved knowledge on their transformation/dissolution and mobility in different aquatic settings [10].

Major challenges to assess the risks of NPs released to the environment include separation of these particles from naturally occurring colloidal particles of similar characteristics, to obtain knowledge on physico-chemical transformations of these particles, and to predict their environmental mobility [11, 12]. Upon their dispersion, their characteristics change in a manner that is particle-specific (*e*.*g*. size distribution, shape, chemical composition, surface charge) and that depends on the surrounding solution chemistry (*e*.*g*. pH, ionic strength, presence of organic matter or other ligands) [13–16].

Differences in surface chemical and structural properties of NPs in aquatic settings are strongly related to their interaction with natural organic matter (NOM) [17, 18]. Humic and fulvic acid, with carboxylic and phenol groups being the most common surface active groups, are the main components of NOM and the most abundant organic matter in environmental settings [19]. Earlier studies have shown that interactions between NOM and NPs in surface water can, depending on *e*.*g*. pH and concentration of humic acid, result in either the breakdown or the expansion of NP agglomerates [20]. In general, de-agglomeration promotes transport of NPs in aquatic environments [21] and particle agglomeration results in the accumulation of NPs adjacent their dispersion source due to sedimentation processes [22]. The extent and rate of dissolution of dispersed NPs influence their mobility and transport to different aquatic settings. Interactions between dispersed NPs and different inorganic and organic ligands, such as NOM, also influence their chemical speciation and hence their toxic potential [18].

There are several studies that focus on the interaction between NOM and nanomaterials such as carbon-based NPs, surface modified NPs, or metal oxide NPs [14, 23–26]. Fewer studies have been performed on bare (non-functionalized) metal NPs. Kim *et al*. report *e*.*g*. that the interaction between humic substances (NOM) and Ag NPs reduces their toxicity towards Japanese medaka embryos [27]. Cu NPs have conversely been found to dissolve more rapidly in the presence of NOM [28]. This was explained by complexation between the Cu NPs and functional groups of NOM, and a concomitant weakening of the surface oxide.

The aim of this study was to fill knowledge gaps related to the fate of environmental dispersion of metal NPs for future input in predictive NP fate models. NPs of copper (Cu), aluminum (Al) and manganese (Mn) have been studied in synthetic freshwater. More specifically, the objectives were to; *i)* elucidate the effect of NOM on particle stability and mobility and NP surface interactions, *ii)* determine the extent of metal dissolution and speciation of the NPs as a function of different types of NOM, and *iii)* contrast the behavior of the metal NPs with commercially available silica NPs (SiO_2_ NPs), used in *e*.*g*. paint formulations, which have totally different physico-chemical and surface chemical characteristics compared with the metal NPs.

NOM is in this study represented by humic acid (HA) and dihydroxy benzoic acid (DHBA). DHBA is a small molecule that represents a degradation product of HA. An improved understanding of the mobility, stability and transformation/dissolution processes upon environmental entry is crucial for accurate future predictions and assessments of the environmental fate of dispersed metal NPs [10].

## Materials and methods

### Nanoparticles

Cu NPs (BET surface area of 7.2 ± 0.1 m^2^/g and a primary size of 50–200 nm), produced via wire explosion, were kindly provided by Assoc. Prof A. Godymchuk, Tomsk Polytechnic University, Russia. Detailed particle characterization is given elsewhere [29, 30, 31].

The Mn NPs (Lot#1441393479–680) of purity 99.9% (BET surface area of 26 ± 1 m^2^/g and primary size of approx. 20 nm) was supplied by American Elements (Los Angeles, CA, USA). Detailed particle characterization is given elsewhere [29, 32].

The Al NPs (BET area of 28.9 ± 1 m^2^/g; primary size of approx. 50 nm) were purchased from NanoAmor (Houston, TX, USA).

The SiO_2_ NPs (specific surface area of 130 m^2^/g, corresponding to a mean particle diameter of 22 nm according to the manufacturer), industrially produced sodium stabilized amorphous silica particles (40 wt-% suspension, Bindzil 40/130), were kindly supplied from Akzo Nobel Pulp and Performance Chemicals AB. A particle suspension of 1 g/L in 10 mM NaCl were prepared.

### Chemicals, reagents and solution preparation

All experimental vessels were acid cleaned in 10 vol.% HNO_3_ for 24 h followed by repeated rinsing with ultrapure water prior to each study. All salts required for preparation of the freshwater (FW) including sodium bicarbonate (NaHCO_3_), potassium chloride (KCl), calcium chloride (CaCl_2_·H_2_O) and magnesium sulfate heptahydrate (MgSO_4_·7 H_2_O), were obtained from Sigma Aldrich (Sweden). Humic acid (HA), extracted from Suwannee River (Suwannee River Humic Acid Standard II), was purchased from the International Humic Substances Society (USA). Dihydroxy benzoic acid (2,3-DHBA and 3,4-DHBA) was purchased from Sigma Aldrich (Sweden).The chemical composition of FW is given in Table 1 and prepared using ultra-pure water (18.2 Mῼ cm resistivity; Millipore, Solna, Sweden) following the standard described in the OECD (Organization for Economic Cooperation and Development) transformation/dissolution protocol [33]. The pH was adjusted to 6.2 using additions of 5 vol.% HNO_3_, with minor effects on the ionic strength of the solutions (<0.1% increase). Redox potential measurements were performed using a Redox ORP electrode (Mettler Toledo InLab).

**Table 1:** Chemical composition of synthetic OECD freshwater (pH 6.2, total ionic strength: 0.5 mM, redox potential: 285 mV).

Salt	Concentration (g/L)	Molar concentration (M)
**NaHCO**_**3**_	0.0065	7.5·10^−5^
**KCl**	0.00058	7.8·10^−6^
**CaCl**_**2**_**·H**_**2**_**O**	0.0294	2·10^−4^
**MgSO**_**4**_**·7H**_**2**_**O**	0.0123	5·10^−5^

2,3-DHBA and 3,4-DHBA were added to FW in two concentrations (0.1 mM [15.42 mg/L] and 1 mM [154.12 mg/L]) and used after an equilibration time period of 24 h. The pH was adjusted to 6.2 before the experiments. HA solutions were prepared by manual mixing dissolving HA powder (1 mg) in 1 mL 0.1 M NaOH. FW was thereafter added to the desired HA concentration (2, 20, 40 mg/L). The solution was left to equilibrate for 48 h followed by pH adjustment to 6.2 prior to the experiments. 20 mg/L corresponds to an upper concentration of HA in surface water [34]. Since the actual molecular mass of HA is unknown and ranges from 1–10 kDa [35], the molar concentration in solution cannot be reported.

### Preparation of metal NP dispersions

6 mg of each NP was weighed in scintillation vials using a microbalance (Mettler-Toledo Ag Model-XP26DR). Ultrapure water (6 mL) was added to obtain a stock solution of 1 g/L that was probe sonicated (Branson Sonifier 250, Ǿ 13 mm, 400 W output power, 20 kHz, 20% amplitude-continuous mode) for 882 s. This resulted in a supplied acoustic energy of 7056 J (calibration procedure described elsewhere [32]). The solution was kept in an ice bath to minimize heating effects. After sonication, the NP dispersion was diluted in FW to a nominal NP concentration of 0.1 g/L. The added concentration was measured and found lower (in the range of 0.03–0.08 g/L) due to rapid sedimentation of NPs in the stock solutions. Differences between the nominal and the added dose of reactive metal NPs are reported elsewhere [32]. Control samples were therefore always analyzed in order to determine the added NP dose, and the dispersions were used immediately after preparation.

### Particle size at dry conditions and in solution

The size and morphology of the metal NPs were investigated at dry conditions by means of transmission electron microscopy (TEM), using a Hitachi HT7700 microscope operating at 100 kV. The NPs were dispersed in butyl alcohol and pipetted onto TEM copper grids coated with holey carbon films (Ted Pella, USA). TEM images were recorded in bright field mode.

The size of the SiO_2_ NPs were studied using scanning electron microscopy (SEM, Supra 60 VP microscope, Zeiss, Germany). The SiO_2_ NPs were deposited on pre-cleaned silicon wafer (5 min sonication in ethanol, acetone, and water, followed by 5 min incubation in an ATC polycation suspension (0.1 wt%).

The size distribution of metal NPs in solution was investigated by means of photon cross correlation spectroscopy (PCCS) using a Nanophox instrument (Sympatec GmbH, Claustal, Germany) using UVette Cuvettes (routine pack, Sympatec GmbH, Claustal, Germany). Changes in particle size were recorded after 0.25, 4, 6 and 24 h of exposure. Triplicate samples were investigated for each time period and the size distributions were obtained based on a non-negative least square (NNLS) algorithm. Presented size distributions reflect integrated single distributions from three replicas plotted using the PCCS software (Windox 5). Mean particle sizes reflect intensity weighed results. The mean count rate of photons was collected over 100 s.

FW with DHBA or HA without any NPs were run in parallel to each experiment. As no signals were observed in any of the background measurements, observed PCCS results elucidate the behavior of the NPs in solution.

The sedimentation velocity (m/s) was calculated according to Eq 1

$$Sedimentation\;velocity=\frac{h}{t}$$ Eq (1)

Where *h* is the height of the medium in the cuvette, and *t* the time for complete particle sedimentation.

The particle size distribution of the SiO_2_ NPs in saline (1 mM NaCl, 0.1 g/L) solutions containing DHBA or HA was measured with dynamic light scattering (DLS) using a Malvern Zetasizer Nano ZS (Malvern, Sweden) instrument with a He-Ne laser (λ = 632.8 nm) over a total time period of 21 days. The solution pH was adjusted with HCl or NaOH to 6.0 ± 0.1. All results are reported as intensity-weighted size distribution.

### Zeta potential

Zeta potential measurements (calculated from the electrophoretic mobility using the Smoluchowski approximation) were performed in triplicate using a Malvern DLS Zetasizer Nano S system (Malvern, Sweden) at a temperature of 25°C and an equilibration time of 300 s before each measurement. The results have been interpreted with emphasis on trends instead of absolute values due to the limitations in determination of the zeta potential in terms of *e*.*g*. rotational diffusion or shape effects, as explained by Bhattacharjee [36]. No contributions from bulk solutions with DHBA or HA without any NPs were observed.

### Surface oxide investigations

The surface oxide composition of the Al NPs was examined using X-ray photoelectron spectroscopy (XPS) using a Kratos AXIS UltraDLD x‐ray photoelectron spectrometer (Kratos Analytical, Manchester, UK) driven by a monochromatic 150 W Al x‐ray source. Detailed spectra (20 eV pass energy) were acquired for carbon C 1s, oxygen O 1s and Al 2p and Al 2s. Surface compositional information of the Cu and the Mn NPs is reported elsewhere [29, 30].

### Surface adsorption of solution ligands

Surface adsorption studies were performed using a Bruker Tensor 37 FTIR spectrometer combined with a Platinum Attenuated Total Reflection (ATR)-Fourier Transform Infrared (FTIR) accessory (Bruker, Sweden). The ATR accessory was equipped with a diamond ATR crystal. The angle of incidence of the IR beam was 45°. Spectra were collected with 4 cm^-1^ resolution and 256 scans were co-added for each spectrum.

The NPs were dispersed as described above, with the difference that the medium was ethanol and that the particle loading was 2.5 g/L in the sonicated stock solution. This dispersion was drop cast (total volume approx. 50 μL) onto the ATR crystal and left to evaporate for 2 h before the measurements. An ATR-FTIR flow accessory was there after placed onto the film and FW was used to rinse the system to remove any loosely attached NPs. A spectrum of the particle film and FW was used as background for the studied NP-solutions of interest. Control experiments confirmed the lack of contribution from bulk species of DHBA and HA in the spectra of adsorbed species, unless noted.

### Metal release

NP suspensions were exposed in an incubator at bilinear shaking conditions (Stuart S180, 25°C, 25 cycles/min) for 0, 4, and 24 h in FW with and without DHBA or HA. After exposure, the DHBA containing FW solutions were filtered using a pore size of 20 nm (Anotop 25, Whatman) to separate remaining particles from released ionic and labile species. FW-solution samples containing HA and NPs were centrifuged for 1 h (Beckman Optima L-90K, SW 28 rotor, 52900 g) to enable particle separation. Centrifugation was the preferred method for particle separation of samples containing HA due to metal adsorption within the membrane upon filtration (effects that were negligible when centrifuging the same samples). Quantification of the loss of metals upon filtration and centrifugation was analyzed by means of atomic absorption spectroscopy (AAS, AAnalyst 800, PerkinElmer, Sweden) on acidified samples (pH<2) using 65% HNO_3_ to ensure measurements of the total metal concentration in the supernatant.

Released metal concentrations were determined by means of AAS in flame mode for Cu and Mn, and in graphite furnace mode for Al. Standards were prepared for each element from calibration standards supplied from PerkinElmer (Stockholm, Sweden). Before analysis, the limit of detection (LOD) was determined [37] for each metal in FW solutions containing DHBA or HA. The LOD was 0.77 mg/L for Cu, 0.53 mg/L for Mn, and 0.02 mg/L for Al. The recoveries for added metal to the respective solutions were in the range between 80 and 100% for all metals.

Standard samples were run every 5^th^ sample for quality control and recalibration made if the drift exceeded 5%. All reported data reflects mean values of triplicate samples with corresponding blank data subtracted.

### Prediction of metal speciation by means of thermodynamic modelling

Solution speciation in FW, FW+2,3-DHBA, and FW+3,4-DHBA were calculated using the Joint expert speciation system (JESS, v. 3.0)[38], based on solution composition (Table 1). Speciation modelling was also performed for Cu, Al, Mn, in FW containing HA using the Visual MINTEQ model (v. 3.0) [39].

## Results and discussion

### NP characteristics

**Fig 1:**
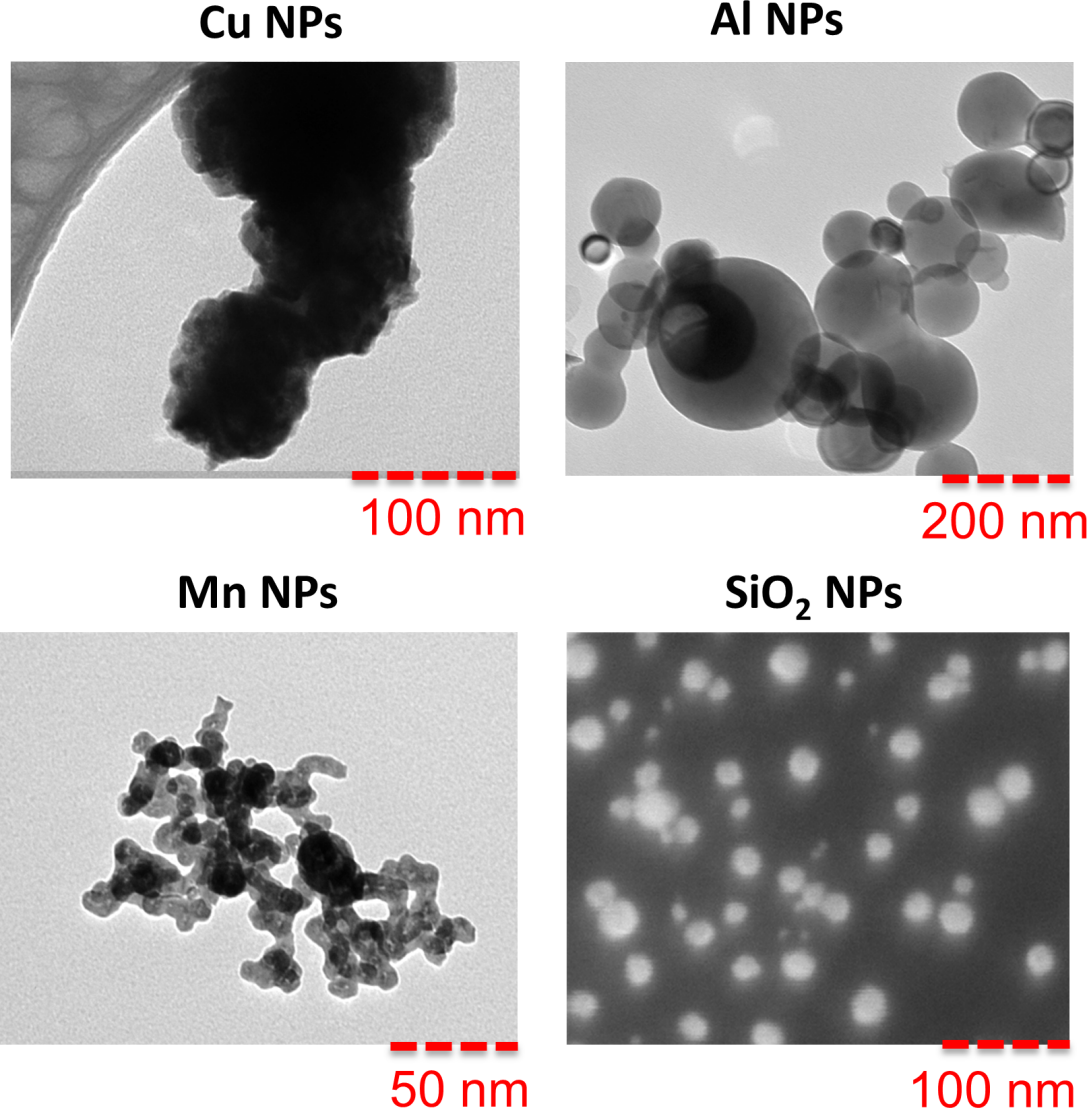
Electron microscopy. Images of Cu, Al, Mn NPs (TEM) and SiO_2_ NPs (SEM).

Electron microscopy images of the Cu, Al, Mn and SiO_2_ NPs are shown in Fig 1. The images clearly show that the Al and SiO_2_ NPs are spherically shaped, while the Cu and Mn NPs are less spherical and also have a rougher surface (Cu NPs). The primary size of the Mn NPs was approximately 20 nm, ca. 50 nm for the Al NPs, and in the range of 20–200 nm for the Cu NPs. SiO_2_ NPs had a primary particle size of 20–30 nm. The specific surface of the metal NPs decreased in the following order 26.1 ± 1 m^2^/g (Mn NPs) > 14 ± 0.7 m^2^/g (Al NPs) > 7.2 ± 0.7 m^2^/g (Cu NPs).

According to previous XPS findings, the surface oxide of the Cu NPs is composed of both Cu_2_O and CuO [30], whereas MnO_2_ and Mn_2_O_3_ are the main surface components of the Mn NPs [29]. The XPS investigation of the Al NPs revealed both a metal (Al 2p_3/2_ peaks at 72.2 eV [40]) and an oxidized peak (74.4 eV), the latter assigned to Al_2_O_3_ [41].

The isolectric point (IEP) in 1 mM NaClO_4_ was determined to pH 3.2 ± 0.6 for the Mn NPs and to pH 6.6 ± 0.7 for the Al NPs [29]. The IEP could not be determined for the Cu NPs due to particle sedimentation.

### Humic acid and DHBA adsorb and coordinate via different functional groups to the surface of the Cu, Mn and Al NPs

**Fig 2:**
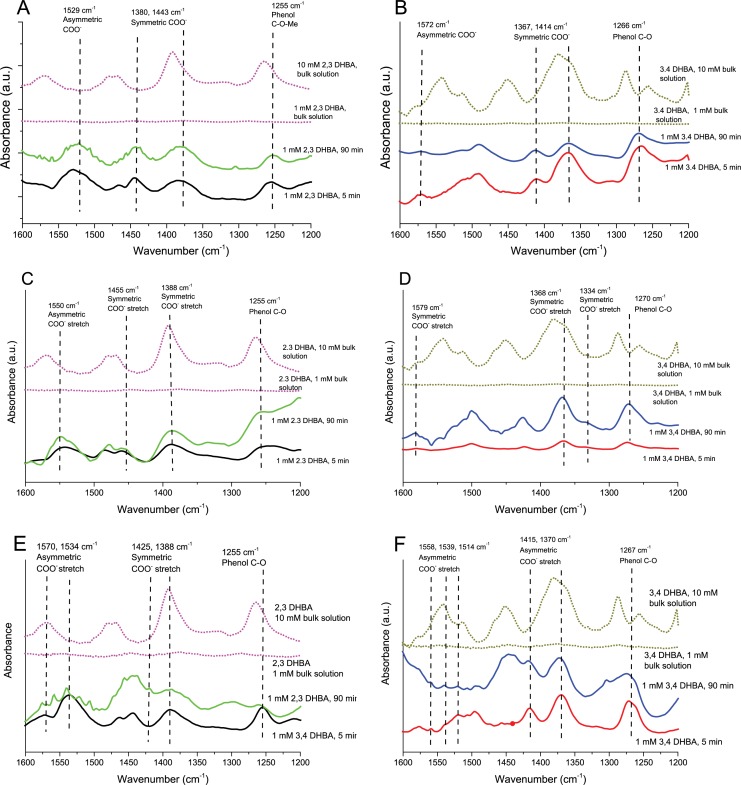
ATR-FTIR spectra in DHBA. ATR-FTIR spectra of metal NPs exposed in freshwater (FW) containing 1 mM DHBA (2,3 and 3,4) at pH 6.2; (A,B) Cu NPs, (C,D) Al NPs, (E,F) Mn NPs. Spectra for solution species of DHBA (without any NPs) are included for comparison (dotted lines) at DHBA concentrations of 1 mM and 10 mM. Main bands for adsorbed DHBA to the NP surfaces are marked in the spectra.

ATR-FTIR was performed to study surface adsorption of DHBA to the Cu, Mn, and Al NPs. The results are presented in Fig 2 as a function of time. Since control experiments at the same DHBA concentration (1 mM DHBA) without any NPs (dotted lines) confirmed the lack of contribution from DHBA species in the bulk solution, observed peaks reflect surface adsorbed DHBA. Spectra from solution species of DHBA, collected at a 10 times higher concentration (10 mM) concentration, are included for comparison. Table 2 shows a compilation of the main peaks observed in the 1200–1600 cm^-1^ region and their corresponding assignments.

**Table 2:** ATR-FTIR peak positions observed in ATR-FTIR spectra of the DHBA isomers adsorbed on the surface of the Cu, Mn and Al NPs, together with their assignments based on the work of Guan et al.[42]). All spectra were collected at a DHBA concentration of 1 mM at pH 6.2, except for the solution spectra of DHBA that were acquired at 10 mM in order to obtain sufficient signals.

Peak positions (cm^-1^)								Peak assignment
2,3-DHBA				3,4-DHBA				
Solution	Cu NPs	Mn NPs	Al NPs	Solution	Cu NPs	Mn NPs	Al NPs	
1568	1529	1570, 1534	1592, 1550	1553, 1513	1572	1558, 1539, 1514	1579	Asymmetric COO^-^ stretch
	1512				1512	1496		Asymmetric COO^-^ / C-C stretch
1481, 1467	1467	1463, 1441	1478, 1454	1464, 1452	1491	1445	1498, 1425	C-C ring stretch
1392	1443, 1380	1425, 1388	1387	1381, 1366	1414, 1367	1415, 1370	1368, 1334	Symmetric COO^-^ stretch
	1255	1255	1254		1266	1267	1270	C-O stretch in complexed phenolic C-O-Me group
				1285				Phenol C-O(H) bending
1264				1255				Phenol C-O(H) stretching

**Fig 3:**
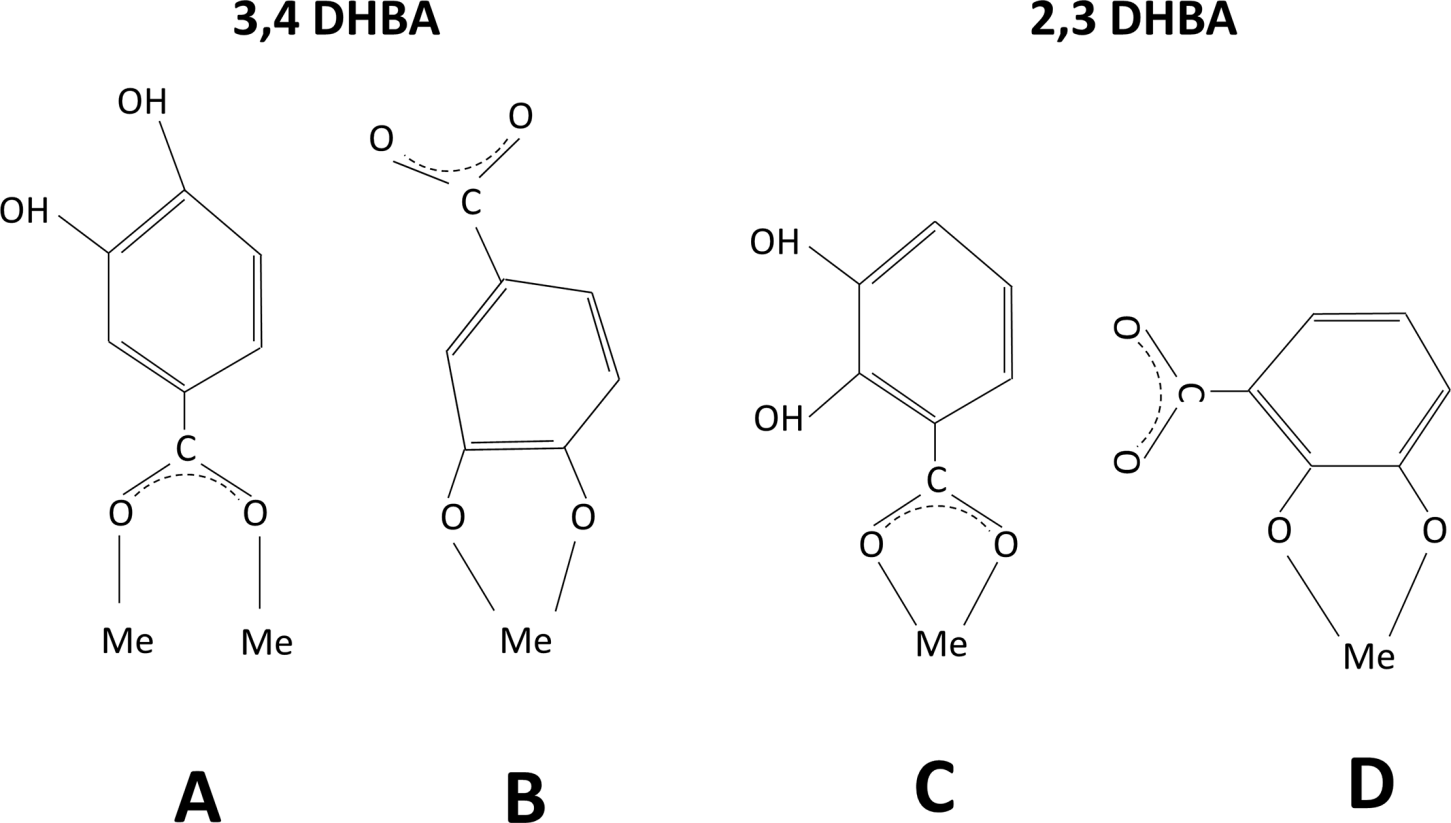
NOM adsorption coordination. Different coordination modes (A-D) between the two DHBA isomers and metal surfaces (Me), as deduced from ATR-FTIR results.

Spectra of adsorbed DHBA species compared with corresponding solution vibrational modes revealed shifts in carboxylate stretches, C-C ring stretches, and phenolic C-O(H) vibrations. Different possible surface coordination modes for the DHBA monomers are illustrated in Fig 3 [42, 43]. For 3,4-DHBA, the phenolic C-O(H) stretch red-shifts 16–20 cm^-1^, which together with the disappearance of the phenolic C-O(H) peak bending mode, suggest a catecholate chelate coordination between 3,4-DHBA and the metal NPs, Fig 3B [42].

The symmetric carboxylate stretch of the 3,4-DHBA molecule is split into two peaks, indicating complexation between the carboxylate group and the metal surface [44]. The asymmetric carboxylate band also splits into two or more bands for the Cu or Mn NPs (but not for the Al NPs). The difference in wavenumber (Δν) between the symmetric and asymmetric carboxylate peaks can provide information on the bonding geometry of the carboxylate group to the metal [44]. Due to a partial overlap of the asymmetric COO^-^ and C-C stretches in the 1500–1600 cm^-1^ region [42], distinct determinations of Δν are not possible. Relatively accurate estimates however can be made and are listed in Table 3. The observed Δν for all metal NPs is comparable to the corresponding Δν for the 3,4-DHBA molecule in FW, which imply bridging mainly via a binuclear bidentate coordination as shown in Fig 3A [42]. Thus, ATR-FTIR findings indicate the co-existence of two kinds of coordination of the adsorbed 3,4-DHBA monomer on all metal NPs, one binuclear bidentate complex and one catecholate type complex.

**Table 3:** Differences between symmetric and asymmetric carboxylate stretch wavenumbers (Δν) for the DHBA monomers in solution and when adsorbed on the metal NPs of this study.

	Δν (cm^-1^)	
Solution	2,3-DHBA	3,4-DHBA
**DHBA+ FW only**	180	≈150
**DHBA+ FW + Cu NPs**	150	≈145–160
**DHBA + FW+ Mn NPs**	150	≈145–160
**DHBA + FW+ Al NPs**	160	≈160

In the case of FW with the 2,3-DHBA monomer and Cu NPs, the phenolic C-O(H) stretching vibration shifted from 1264 cm^-1^ to 1255 cm^-1^. Analogously to findings for FW with the 3,4-DHBA monomer, this suggests the formation of a catecholate type binding between the phenolic C-O group and the Cu NP surface (Fig 3D). In addition, upon adsorption of 2,3-DHBA, the symmetric COO^-^ stretching vibration shifted from 1391 cm^-1^ to 1380 cm^-1^ and the asymmetric from 1568 cm^-1^ to 1529 cm^-1^ (Table 2). A reduced Δν compared with the 2,3-DHBA monomer in solution indicates the formation of a mononuclear bidentate surface complex (Fig 3C).

The C-O(H) peak observed for the Mn NPs shifted to lower wavenumbers in the case of FW with the 2,3 DHBA monomer, again indicating a catecholate chelate surface complex. The shift in carboxylate stretches indicates a mononuclear bidentate surface complex, analogous to observations made for the Cu NPs.

**Fig 4:**
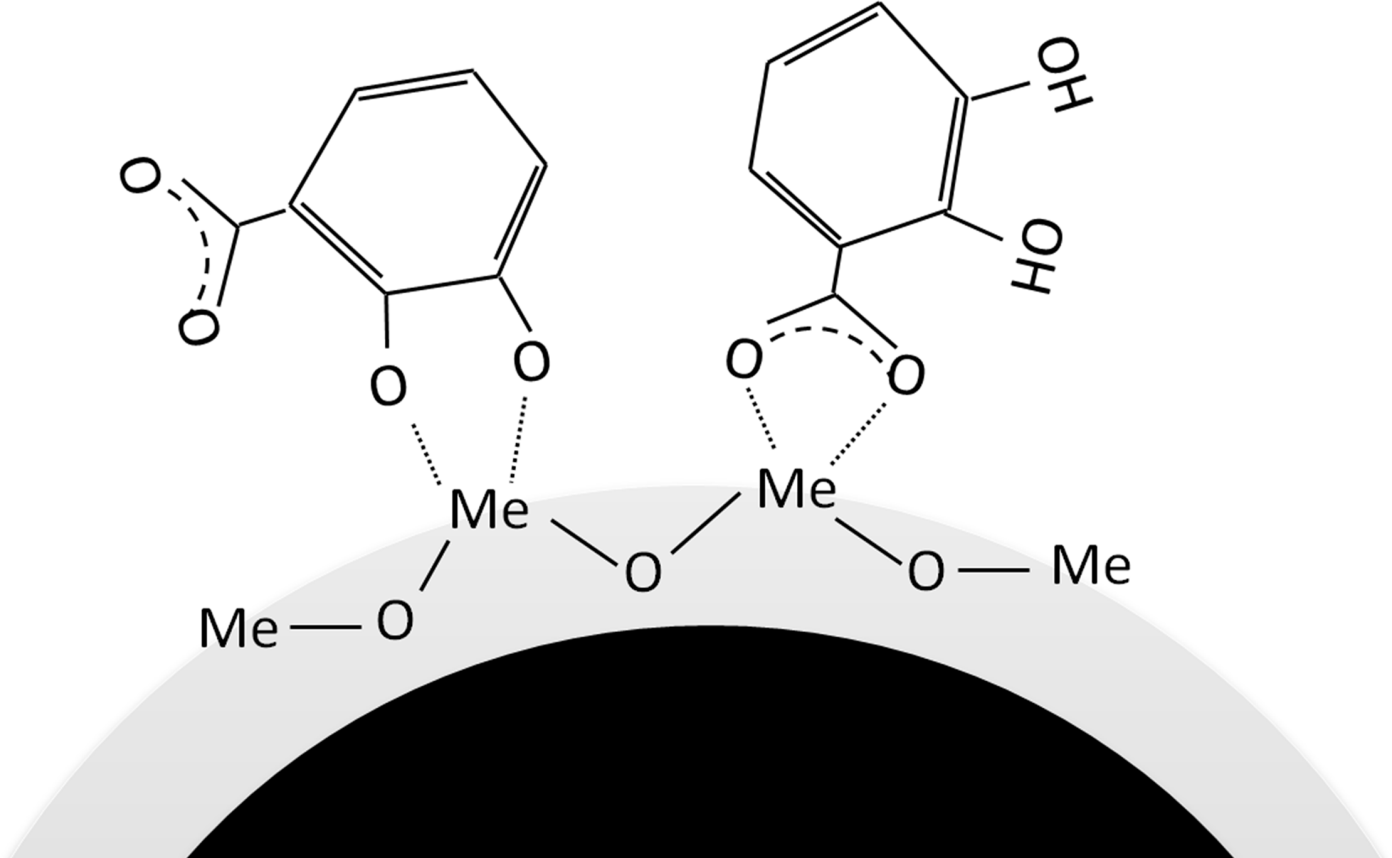
DHBA adsorption. Schematic illustration on the adsorption of DHBA (here 2,3-DHBA) to a given metal NP (with a surface oxide of metal “Me”) in freshwater as deduced from ATR-FTIR findings.

For the Al NPs, no shift in the phenol C-O(H) stretch was observed in FW containing the 2,3-DHBA monomer, which implies the lack of involvement of the phenol groups in its surface coordination with the Al NPs. The Al NPs revealed significant changes over time (5 and 90 min) in terms of relative strengths of the different vibrational bands, not observed for the Cu and the Mn NPs, which indicate slower adsorption over time. Similarly to Cu NPs, observed differences in Δν between bulk solution and adsorbed species indicate mononuclear bidentate coordination. Fig 4 depicts a schematic image of how DHBA adsorption (exemplified for the 2,3-DHBA monomer) on a given metal NP might occur.

**Fig 5:**
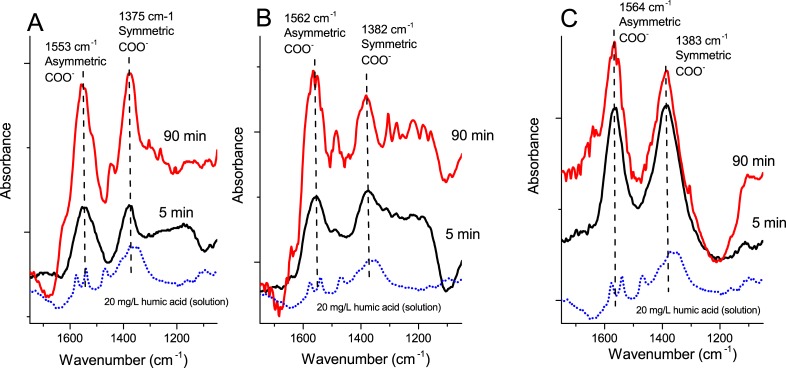
ATR-FTIR spectra in humic acid. ATR-FTIR spectra for the different metal NPs in synthetic freshwater(FW) containing 20 mg/L HA, pH 6.2; (A) Cu NPs; (B) Al NPs and (C) Mn NPs.

Fig 5 shows the ATR-FTIR spectra of the Cu, Al and Mn NPs exposed in FW containing 20 mg/L HA at pH 6.2. A solution spectrum of 20 mg/L HA is included for comparison. The results show evident changes in the spectra (peaks shifts and new peaks) for the metal NPs compared to peaks corresponding to HA in solution. After 5 min, broad peaks, centered at 1550 ± 10 cm^-1^ and 1375 ± 10 cm^-1^, dominated the spectra for the Cu, Al, and Mn NPs. These modes correspond to asymmetric and symmetric stretching vibrations of carboxylate, respectively [45]. The observed shifts (approx. 15 cm^-1^ for the symmetric stretch) compared with the solution spectra indicate coordination between carboxylate groups of HA and metal atoms of the surface oxides of the NP surface. These modes are relatively broad (full width half maxima of 70–100 cm^-1^) compared with *e*.*g*. the corresponding peaks for adsorbed DHBA (approx. 35 cm^-1^). The broadening is a result of different kind of carboxylate groups that give rise to peak shifts as a result of the presence of different polar groups in their vicinity, or similar groups with different coordinations to the surface,. This effect is expected since HA has a largely heterogeneous chemistry [45].

The absorbance of the vibrational bands increased over time (5 min compared with 90 min), which implies an increased adsorption of HA to the surface, possibly as multilayers. A large number of additional peaks became evident after 90 min compared with 5 min. As HA contains many different chemical groups, detailed assignments were not possible beyond the assignment of carboxylate groups as mentioned above. Observed changes in the 1400–1650 cm^-1^ region may imply interactions with different types of carboxylates than observed after 5 min exposure. New bands occurring after 90 min could possibly originate from chemical groups not directly attached to the surface (*e*.*g*. as a part of a multilayer), as this would cause a shift with respect to the surface-bound groups. Observed bands at 1000–1400 cm^-1^ may originate from *e*.*g*. phenolic groups C-O(H) (as in the case of DHBA), quinines, or alkenes [46].

Weak adsorption of DHBA and HA onto SiO_2,_ mainly due to strong electrostatic repulsion due to the higher negative charge of silica NPs compared to metallic NPs [47] and issues with film stability prevented collection of meaningful spectra in the silica system. In all, the ATR-FTIR spectral analysis of Cu, Mn, and Al NPs interacting with the DHBA monomer or HA in synthetic FW indicates multiple surface coordination between NOM and the NPs, all via inner sphere complexes (covalent bonding with surface metals)[48] as deduced from vibrational band shifts of carboxylates and phenolic C-O groups and differences between peak frequencies of symmetric and asymmetric carboxylate stretches.

### The zeta potentials of the metal NPs changed upon NOM adsorption and were related to the conformation pattern of adsorbed NOM

**Fig 6:**
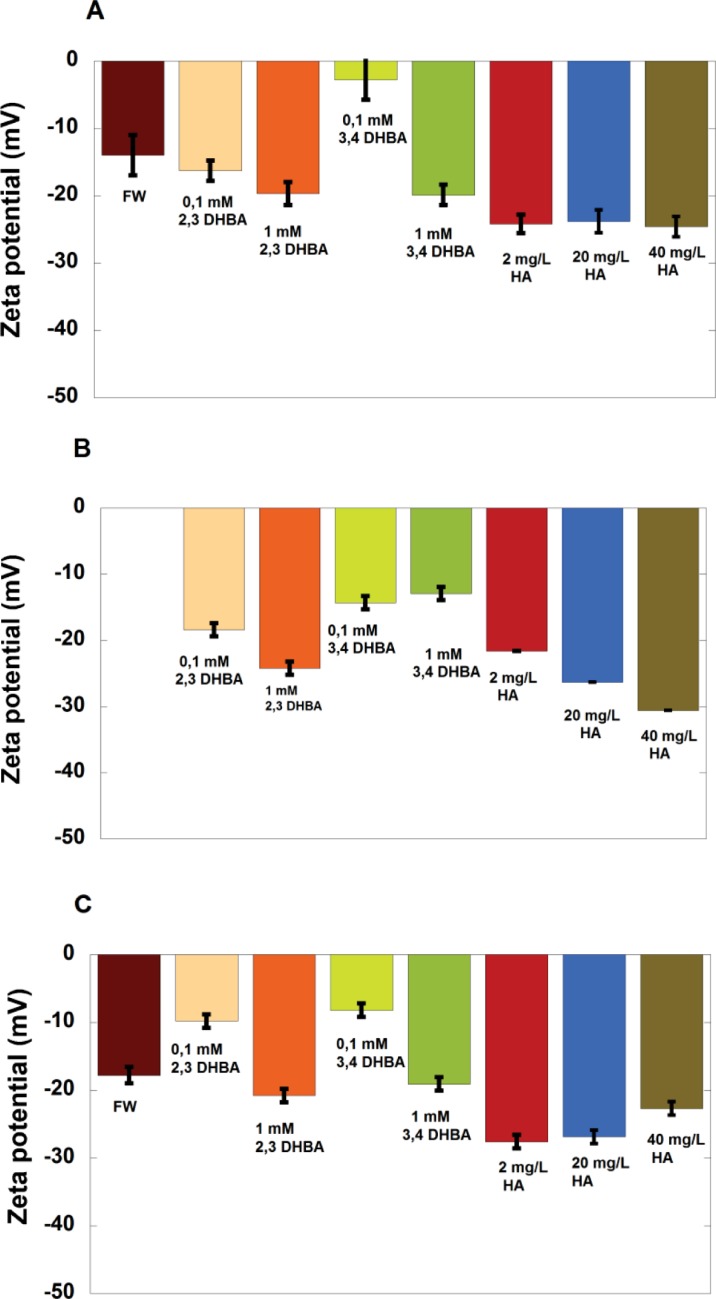
Zeta potential. Zeta potential determined for the different metal NPs in freshwater (FW) containing DHBA or humic acid (HA), measured approx. 15 min after solution sonication; (A) Cu NPs, (B) Mn NPs and (C) Al NPs. The error bars represent one standard deviation, derived from three independent samples.

The influence of DHBA or HA in FW on the measured zeta potential of the NPs is illustrated in Fig 6.

For the Cu NPs (Fig 6A), the zeta potential became slightly more negative in FW containing the 2,3-DHBA monomer (0.1 and 1 mM) and the 3,4-DHBA monomer (1 mM) compared to Cu NPs in FW only. This is in line with exposure of negatively charged (dissociated) carboxylic groups facing the aqueous phase (FW) as shown by ATR-FTIR (Figs 2 and 5) and illustrated in Fig 3B for the 2,3-DHBA monomer. In contrast, the measured zeta potential of the Cu NPs was less negative in 0.1 mM 3,4-DHBA compared with conditions in FW only. Adsorption of the 3,4-DHBA molecule can, at these lower concentrations, hence neutralize the surface charge of the metal NPs, an effect that may be related to differences in preferred coordination with the surface at higher concentrations (1 mM) of 3,4-DHBA and 2,3-DHBA. The zeta potential is measured at the outermost interface between the adsorbed layer and the aqueous phase. A less negative potential might hence be explained by a different orientation of the adsorbed DHBA molecule coordinated to the Cu NP surface, *i*.*e*. a preferred coordination of the negatively charged carboxylic group towards the surface (carboxylate bonding) exposing more uncharged phenolic groups towards the aqueous (FW) phase. At higher concentration of 3,4-DHBA and in 2,3-DHBA in FW, a higher degree of catecholate bonding exists rendering in a more negatively charged (COO^-^) interface.

The measured zeta potential for the Cu NPs in presence of HA at pH 6.2 also became significantly more negative compared with FW only, *i*.*e*. indicative of surface adsorption of HA (confirmed by ATR-FTIR results, see Fig 5). This is in agreement with previous studies of TiO_2_ and Al_2_O_3_ NPs in the presence of HA [49, 50]. HA is highly negatively charged in 1 mM NaCl solutions (between pH 3 to 11) in which it adopts a semi-rigid random coil structure [49]. Upon surface adsorption on the metal NPs, the plane of charge will move outwards, and the measured zeta potential will be determined by the outermost part of the adsorbed layer of HA.

The lack of concentration dependence in the presence of humic acid for the Cu NPs indicates that the zeta potential at the plane of charge is independent of the layer thickness of humic acid (not accessible by zeta potential measurements). The same trend as seen for the Cu NPs was observed for the Al NPs, except in the case of FW with 2,3-DHBA of the lower concentration, 0.1 mM (Fig 6C). Here the magnitude of the zeta potential was lower compared to FW, which indicates a lower degree of catecholate coordination between the 2,3-DHBA molecules and the Al NP surface.

No reliable determination of the zeta potential was possible for Mn NPs in FW only due to their rapid sedimentation. However, in the presence of DHBA or HA in FW, the measured zeta potential was negative and its numbers similar as observed for the Cu and Al NPs (Fig 6B). The magnitude of the zeta potential increased with increased concentration of HA, and the zeta potential was less negative in the presence of 3,4-DHBA compared with 2,3-DHBA.

One plausible explanation for the different HA concentration dependence of the zeta potential of the Cu, Mn, and Al NPs may be that the surface oxide of the Mn NPs (IEP = 3.2 ± 0.6 in 1 mM NaClO_4_) is more acidic compared to the surface oxide on the Cu NPs (IEP close to neutral in 1 mM NaClO_4_)[29] and Al NPs (IEP = 6.6 ± 0.7 in 1 mM NaClO_4_) and that this results in a higher electrostatic repulsion between the Mn NPs and HA at lower concentrations (2 mg/L compared with 20 and 40 mg/L) at pH 6.2, *i*.*e*. a thinner and/or less dense adsorbed layer. The increase in negative zeta potential with HA concentration indicates an increased adsorption of HA. Future studies using the quartz crystal microbalance technique with dissipation (QCM-D) may address these issues further.

### The presence of DHBA or humic acid in freshwater delays the extent of agglomeration and sedimentation of the metal NPs, but have no effect on the stability of the SiO_2_ NPs

**Fig 7:**
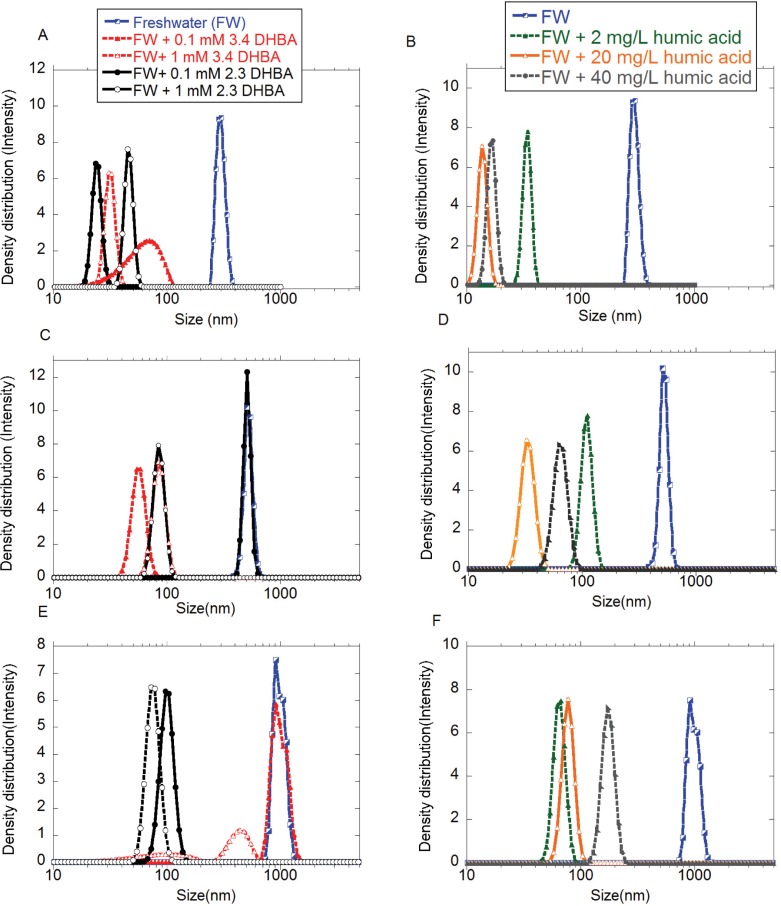
Particle size distribution. Particle size distributions of (A, B) Cu NPs, (C, D) Al NPs, and (E, F): Mn NPs in freshwater (FW) with and without 2,3-DHBA, 3,4-DHBA, or humic acid (HA). Measurements were conducted after approx. 15 min immersion. The pH was 6.2 and the added particle loadings 0.035–0.045 g/L for the Cu NPs, 0.05–0.06 g/L for the Al NPs, and 0.03–0.05 g/L for the Mn NP.

Fig 7 shows particle size distributions in solution for the Cu, Al, and Mn NPs in FW with DHBA (0.1 or 1 mM), or HA (2, 20, or 40 mg/L). The results were obtained after 15 min immersion in the respective solution. The NP loading varied somewhat and was significantly lower than the nominal concentration (0.1 g/L) due to rapid sedimentation already in the sonicated stock dispersion. Differences in nominal and added doses of rapidly agglomerating and sedimenting metallic NPs are described elsewhere [32]. The added dose was in this study 55–65% lower than the nominal dose (0.035–0.045 g/L) for the Cu NPs, 40–50% (0.05–0.06 g/L) for the Al NPs, and 50–70% (0.03–0.05 g/L) for the Mn NP. However, expected concentrations of most NPs in the environment are even lower (<<0.1 mg/L) [8, 51].

The results in Fig 7 indicate that the presence of HA in FW reduces the hydrodynamic sizes of the metal NP-agglomerates with a factor of 5–10. Steric repulsion between adsorbed HA molecules has been shown to induce particle de-agglomeration for hematite NPs [14]. This phenomena was also observed for the Cu and Al NPs in this study (Fig 6) with particle sizes close to their primary sizes (approx. 20 nm for the Cu NPs, and 30 nm for the Al NPs (Fig 1)). The Mn NPs are an exception here with a resulting agglomerate size different from the primary size (approx. 80 nm size compared with approx. 25 nm). This may be connected to the lower affinity of Mn with respect to HA, compared with Cu and Al [52].

In FW containing the DHBA monomer, the agglomerate size was in general reduced, in particular evident for the Cu NPs. These trends were though less obvious than observed for HA with only some agglomerates of similar size as compared with the conditions in FW only. Agglomeration of the Cu NPs in FW with 0.1 mM 3,4-DHBA (broad peak in Fig 7A) correlated well with a lower zeta potential compared with corresponding findings in FW and HA (Fig 6A). Such conditions will significantly reduce any electrostatic stabilization between negatively charged particles. It should be stressed that very high van der Waals attraction between metallic NPs due to their dielectric properties [32] remain even though the adsorption of an organic layer (molecules) changes the dielectric properties of the surface and thereby reduce the magnitude of this attraction.

**Fig 8:**
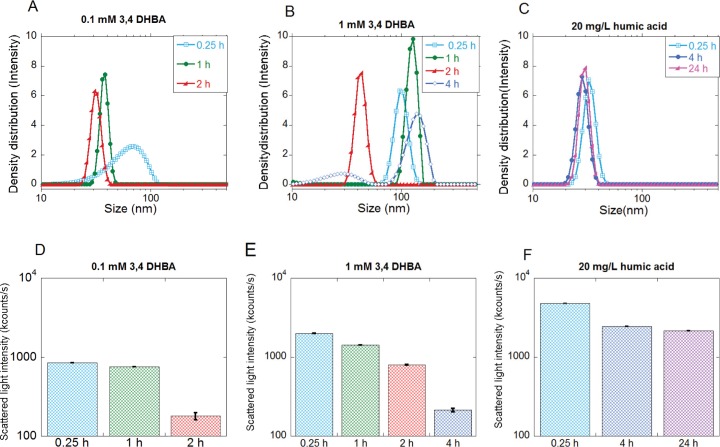
Particle size distribution. Particle size distributions of Cu NPs in (A) FW + 0.1 mM 3,4-DHBA, (B) FW + 1 mM 3,4-DHBA and (C) FW + 20 mg/L HA, and corresponding changes in scattered light intensities (D-F). FW denotes freshwater and HA humic acid.

Essentially complete sedimentation due to rapid agglomeration was evident for all NPs within less than 15 min in FW only and within 1–6 h in FW containing the DHBA monomer. As an example, most Cu NPs sedimented out from solution within 4 h in FW with 0.1 mM DHBA and within 6 h in FW with 1 mM 3.4 DHBA. The size distributions in Fig 8 show the time points before complete sedimentation of the Cu NPs: after 2 h (0.1 mM 3,4-DHBA) and 4 h (1 mM 3.4 DHBA). The Cu NPs were notably more stable in HA (20 mg/L).

Sedimentation velocities of the NPs are given in Table 4, calculated from the time points when the count rates of the scattered light in the PCCS measurements reached similar levels as the background (solutions without any NPs).

**Table 4:** Calculated sedimentation velocities of the Cu, Al and Mn NPs in freshwater (FW), with and without different concentrations of the DHBA monomers or humic acid (HA) based on PCCS findings.

	Sedimentation velocity [m/s]		
Medium	Cu NPs	Al NPs	Mn NPs
**Freshwater (FW)**	3.2 ± 2.1·10^−6^	3.2 ± 2.1·10^−6^	3.2 ± 2.1·10^−6^
**1 mM 2,3 DHBA**	3.2 ± 2.1·10^−6^	1,0 ± 0.3·10^−6^	7.3 ± 1.9·10^−7^
**0.1 mM 2,3 DHBA**	7.3 ±1.9 10^−7^	3.2 ±2.1·10^−6^	7.3 ± 1.9·10^−7^
**1 mM 3,4 DHBA**	4.7 ± 0.8·10^−7^	7.3 ± 1.9·10^−7^	7.3 ± 1.9·10^−7^
**0.1 mM 3,4 DHBA**	5.2 ± 1.5·10^−7^	3.2 ± 2.9·10^−6^	7.3 ± 1.9·10^−7^
**2 mg/L HA**	7.3 ± 1.9·10^−7^	<1.2 ± 0.5·10^−7^*	<1.2 ± 0.5·10^−7^*
**20 mg/L HA**	<1.2 ± 0.5·10^−7^*	<1,2 ± 0.5·10^−7^*	<1.2 ± 0.5·10^−7^*
**40 mg/L HA**	7.3 ± 1.9·10^−7^	6.94 ± 1.8·10^−7^	<1.2 ± 0.5·10^−7^*

* estimated from the observation of particle sedimentation after 24 h, which was incomplete.

The numbers given in Table 4 are hence a conservative estimate of the sedimentation velocities. As seen in Table 4, the sedimentation velocities of the metal NPs were generally reduced according to the following sequence: FW > DHBA > HA. This is in concordance with the observed size distribution findings of particle agglomerates presented in Figs 6 and 7. Calculated sedimentation velocities are in the order of magnitude as expected for agglomerating NPs, *i*.*e*. rapid sedimentation for particle agglomerates sized several hundred nm or more [53]. Compared with recent findings for tungsten carbide NPs exposed to FW containing HA [54], calculated velocities are somewhat lower for the NPs of this study. This may be a result of different effective densities of the NPs (lower effective density leads to slower sedimentation).

It is evident that the steric repulsion, cross-linking, *i*.*e*. bridging of the carboxylic groups by divalent ions (*e*.*g*. Ca^2+^) and/or hydrogen bonding that originate from adsorbed HA plays an important role for the stabilization or destabilization of the NPs. This is for instance evident from the significantly higher sedimentation velocities observed (Table 4) for the Cu NPs at the lowest (2 mg/L) and the highest (40 mg/L) concentration of HA compared with the intermediate concentration (20 mg/L). The steric repulsion must be important since the zeta potentials are similar for the three concentrations of HA (Fig 6). Similar observations were earlier reported by Ghosh *et al*. [50], whom reported that an increase in HA concentration resulted in an entanglement of adsorbed HA molecules. Schneckenburger *et al*. have previously reported cross linking esters and ethers between HA molecules for concentrations of 40 mg/L [55]. Steric repulsion has previously been reported as an important factor for particle stabilization of TiO_2_ NPs in contact with HA [56]. The trend of reduced stability at higher HA concentrations was however not the case for the Al and Mn NPs, which both were relatively stable in terms of size during the 24 h exposure period.

**Fig 9:**
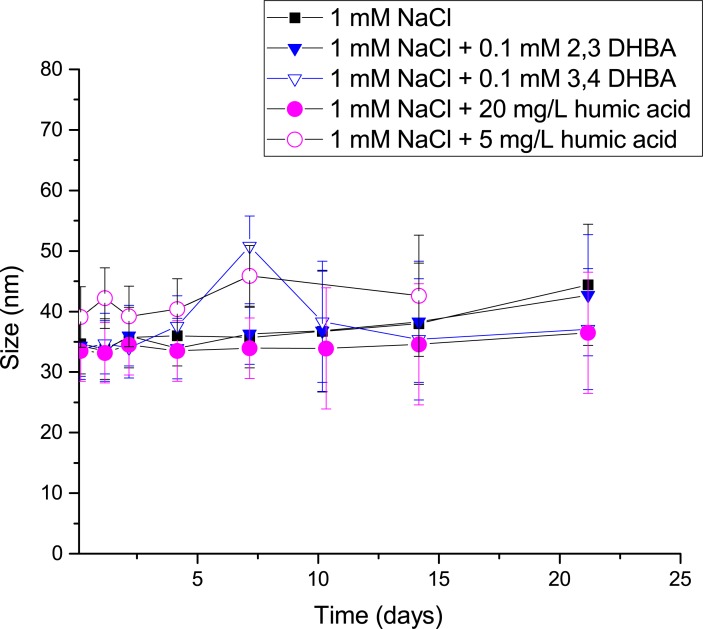
Particle size distribution of SiO_2_ NPs. Average particle size of SiO_2_ NPs over time in 1 mM NaCl with and without the DHBA monomers (0.1 mM) or humic acid (HA) (5 and 20 mg/L). All experiments were performed using a particle concentration of 0.1 g/L.

Investigations were also performed using SiO_2_ NPs in order to explore whether organic matter also would influence the stability of other NPs such as non-metallic particles of different characteristics (Fig 9).

These NPs proved to be very stable in dilute NaCl solutions (of similar ionic strength and pH as FW, *i*.*e*. 1 mM and pH 6.0). A particle suspension free of organic matter was shown to be stable over extended time periods, and the presence of 0.1 mM of either isomer of DHBA or HA (5 and 20 mg/L) in the NaCl solution had a negligible effect on the particle size distribution (Fig 9), *i*.*e*. no agglomeration or sedimentation took place within the 21 day time frame. In contrast to the metal NPs that readily sediment close to their dispersion source, the SiO_2_ NPs are more mobile and could, if dispersed, in a given environmental exposure scenario, potentially be transported to other environmental aquatic settings.

### The presence of NOM (DHBA or humic acid) in freshwater enhances the extent of metal release and changes the metal speciation in solution for released Cu and Al, but show no significant effects on released Mn

**Fig 10:**
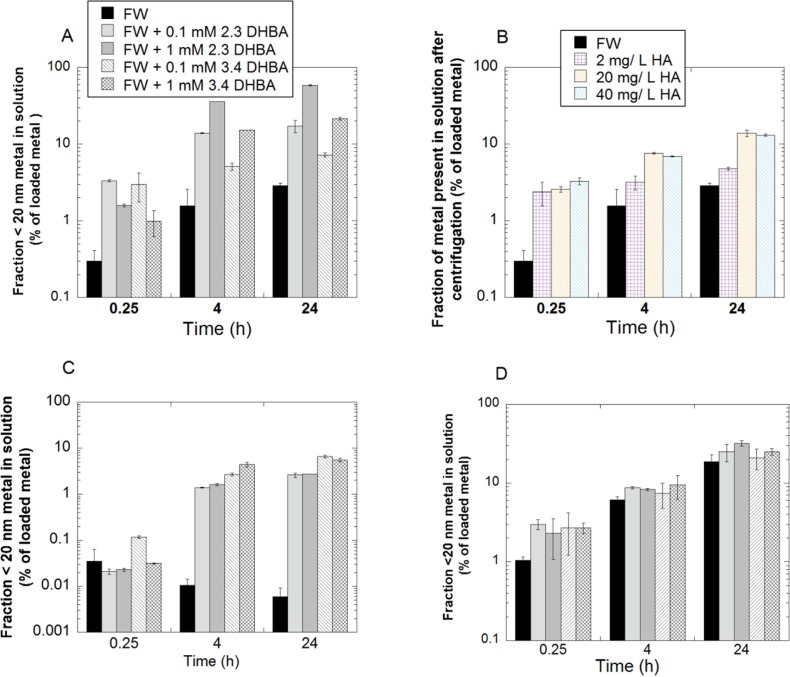
Metal release. Released fraction of metals in solution (after membrane filtration–DHBA; or centrifugation—HA) for (A) Cu NPs in FW+DHBA, (B) Cu NP in FW+HA, (C) Al NPs in FW+DHBA, and (D) Mn in FW+DHBA. All measurements were conducted at pH 6.2. The error bars represent one standard deviation, derived from three independent samples. Note–logarithmic scales on all y-axes. FW denotes fresh water and HA humic acid.

The influence of the presence of DHBA on the extent of metal release was investigated for all metal NPs, while the influence of the presence of HA was investigated for Cu NPs only. The results are presented in Fig 10. In all cases, the presence of DHBA monomers resulted in significantly larger amounts of released metal species (small enough to pass through a 20 nm pore size membrane, *i*.*e*. not particles) in solution compared with FW only. The largest enhancement of metal release was observed for Cu NPs, where the presence of either DHBA isomer increased the release rate by roughly one order of magnitude (Fig 10A). The presence of HA also had a major effect on the release rate from Cu NPs (Fig 10B). The dissolution pattern for the Cu NPs in FW containing HA was similar to observations made for FW+DHBA, and of the same order of magnitude as literature findings [28]. However, an increased HA concentration did not always result in an increased release rate, as seen in the lack of difference between 20 and 40 mg/L HA.

For Al NPs, there are only minor differences at the first data point (0.25 h), but with time two different trends emerge (Fig 10C). In FW, the amount of aluminum in solution decreases with time, while in the presence of DHBA the amount of aluminum in solution increases with time throughout the experiments (*i*.*e*. 24 h). For Mn NPs, the presence of DHBA appears to increase the initial release rate, resulting in higher amounts of released manganese in solution at 0.25 h, while there is negligible impact of DHBA after longer times (Fig 10D).

Increased amounts of released Cu and Al in solution in the presence of NOM are in agreement with the ATR-FTIR results presented in Figs 2 and 5. The coordination of DHBA and HA to the NP surface can weaken the bonds between the metal and oxygen in the surface oxide, with increased metal release as a consequence [28, 48]. As seen in the ATR-FTIR results of the Cu NPs (Fig 2), the 2,3-DHBA molecule was more prone to form mononuclear surface complexes, known to accelerate metal dissolution from metal/metal oxide surfaces more compared with binuclear complexes [48]. This effect may be reflected in the release data for the Cu NPs exposed in FW with significantly more copper released in the presence of 2,3-DHBA compared with the 3,4-DHBA molecule.

Apparently, the carboxylate and phenol groups coordinate with the Al NP surface in a way that promotes dissolution. Conversely, no large difference in metal release rates was observed for the Mn NPs in FW with and without DHBA, except for a few data points at 0.25 h. Even though the DHBA monomer readily adsorbs to the surface, as based on ATR-FTIR findings (Fig 2), this coordination and possible oxide weakening were not rate limiting steps in the dissolution process. Similar observations have been made for Mn NPs in other media, *e*.*g*. cell media containing proteins [29].

Increased NP dissolution may also be a result of increased surface corrosion due to a reduced surface oxide stability induced by the adsorbed NOM, or by a reduced surface pH [57, 58].

When modelling the metal release data using a first order dissolution rate constant, as proposed by Quik *et al*.[59], a half-life in the order of 7–60 h was obtained for the NPs. This underlines the limited mobility of these NPs under given aquatic conditions.

Speciation modelling of released metals in solution was performed using JESS at given concentrations of 1 mg/L in FW, with and without DHBA (Table 5). Speciation predictions using the Visual MINTEQ software were in addition performed for released Cu, Al, and Mn in FW containing HA (2 and 20 mg/L), Table 6.

**Table 5:** Solution speciation of Cu, Al, and Mn in freshwater (FW) with and without the DHBA monomer predicted using the JESS model. The metal concentration in solution was set to 1 mg/L and the pH to 6.2.

	Cu	Al	Mn
**FW**	45% Cu(OH)_2_(s) 41% Cu^2+^ 8% Cu^1+^	100% Al(OH)_3_(s)	100% Mn^2+^
**FW+** **0.1 mM 3,4-DHBA**	100% Cu_2_(3,4-DHBA)_2_	60% AlOH(3,4-DHBA)^-^ 38% Al(3,4D-HBA) 1% Al(3,4-DHBA)_2_^−3^	100% Mn^2+^
**FW+1 mM 3,4-DHBA**	100% Cu_3_(3,4-DHBA)_2_^2+^	55% AlOH(3,4-DHBA)^-^ 35% Al(3,4-DHBA) 8% Al(3,4-DHBA)_2_^3-^	100% Mn^2+^
**FW+0.1 mM 2,3-DHBA**	100% Cu(2,3-DHBA)_2_^2-^	82% Al_2_H(2,3-DHBA)_2_^+^ 7% AlH_2_(2,3-DHBA)_2_^-^ 5% AlH(2,3-DHBA)^+1^	100% Mn^2+^
**FW+1 mM 2,3-DHBA**	100% Cu(2,3-DHBA)_2_^2-^	63% AlH_2_(2,3-DHBA)_2_^-^ 27% Al_2_H(2,3-DHBA)_2_^+^ 6% AlH(2,3-DHBA)_2_^+1^	100% Mn^2+^

**Table 6:** Fraction of released Cu (0.1, 1, 100 mg/L) in solution that forms complexes with humic acid (HA) (1, 20 mg/L) predicted using the Visual MINTEQ software. The metal binding type (covalent, electrostatic) is also given.

Cu (mg/L)	20 mg/L HA	2 mg/L HA
**0.1**	98.6% Cu-HA, covalent 0.2% Cu^2+^	83.2% Cu-HA, covalent 14.6% Cu^2+^ 0.7% CuOH^+^
**1**	94.6% Cu-HA, covalent 4.5% Cu^2+^	60.5% Cu^2+^ 34.5% Cu-HA, covalent 2.8% CuOH^+^
**100**	96% CuOH(s) 2.5% Cu-HA, covalent 1.4% Cu^2+^	98.0% CuOH(s) 1.4% Cu^2+^ 0.5% Cu-HA, covalent
**Mn (mg/L)**		
**0.1**	88.8% Mn^2+^ 7,9% Mn-HA, covalent 2,6% Mn-HA, electrostatic	98.2% Mn^2+^ 0.8% Mn-HA, covalent 0.6% MnSO4 (aq)
**1**	92,3% Mn^2+^ 5,0% Mn-HA, covalent 1,3% Mn-HA, electrostatic	98.2% Mn^2+^ 0.8% Mn-HA, covalent 0.6% MnSO4 (aq)
**100**	98.0% Mn^2+^ 1,3% Mn-HA, covalent 0.2% Mn-HA, electrostatic	99.4% Mn^2+^ 0.4% MnSO4 (aq) 0.1% Mn-HA, covalent
**Al (mg/L)**		
**0.1**	99.4% Al-HA, covalent 21.6% Al(OH)_3_(s)	92% Al(OH)_3_(s) 7.6% Al-HA, covalent
**1**	99,2% Al(OH)_3_(s) 0.8% Al-HA, covalent	99% Al(OH)_3_(s) 0.8% Al-HA, covalent
**100**	99,9% Al(OH)_3_(s) 0.1% Al-HA, covalent	99,9% Al(OH)_3_(s) 0.1% Al-HA, covalent

The results show that released Cu and Al in FW at low concentrations (<0.1 mg/L) and for a realistic concentrations (2–20 mg/L) of HA, relevant for a dispersion scenario [8], predominantly form complexes with HA and DHBA. This complexation substantially reduces the bioavailable fraction in solution (usually attributed to free ions and labile complexes [60]). The speciation prediction of Cu is in line with literature findings [28].

Mn on the other hand has a lower affinity to NOM, which is in line with previous metal release results that show that NOM had no significant effect on the release of Mn from Mn NPs, From this follows that released ions of Mn will remain in a more bioavailable form. Note however that adsorption of metal to colloids naturally present in FW (*e*.*g*. organic or metal oxides colloids) has not been considered here. Such considerations would further reduce the bioavailable fraction of the metal, as metals bound to natural colloids are not regarded as labile.

The increased solubility with the addition of NOM seen for Cu and Al (Tables 5 and 6) will contribute to the increased amounts of released metal into solution (Fig 10). This motivates caution when comparing FW with FW + NOM dissolution rates for the studied NPs, as a solution saturated with soluble metal may appear to show faster dissolution kinetics compared with more realistic, non-saturated conditions.

The observed amounts of released Al in solution from the Al NPs were very low in FW, corresponding to approx. 0.003% of the added particle mass after 0.25 h (Fig 10). This amount was even lower (0.0008%) after 24 h, which suggests precipitation of released Al. The trend with initially increased amounts of dissolved metals into solution followed by their subsequent reduction (due to precipitation/sedimentation of agglomerates/complexes) has previously been observed for other NPs and is due to chemical reactions and changes in particle size and surface curvature over time [61]. Observed findings with reduced amounts of Al in solution are supported by the speciation modelling results shown Table 5.

## Conclusions

A systematic laboratory investigation has been conducted to fill knowledge gaps related to the environmental fate of bare metal NPs of different surface reactivity in a tentative exposure scenario upon dispersion in freshwater (FW) with and without the presence of natural organic matter (NOM). The following conclusions can be drawn;

- Both the DHBA monomers and humic acid in FW readily (< 1 min) adsorbed via multiple coordination on the bare metal NPs (Cu, Mn, and Al).
- Despite the surface adsorption of NOM, particle agglomeration was significant due to strong van der Waal attraction between the metal NPs. The concentration and type of NOM influenced to some extent the degree of particle agglomeration, sedimentation, and dissolution of the metal NPs.
- DHBA was at concentrations of 0.1 and 1 mM unable to stabilize the metal NPs for time periods longer than 6 h, with significant sedimentation as a result. Humic acid in a concentration of 20 mg/L (realistic for surface waters) was on the other hand able to disperse the NPs more efficiently (>24 h). No influence of NOM on the particle stability of the SiO_2_ NPs was observed.
- Al and Cu NPs agglomerated and sedimented readily in freshwater containing both 2 mg/L and 40 mg/L humic acid, whereas Mn was more stable in terms of agglomeration for all concentrations of humic acid.
- The presence of natural organic matter (DHBA or humic acid) largely influences the extent of metal release from Cu and Al NPs (≥10% of the total mass after 24 h). Speciation calculations of released metals into solution (non-sedimented) showed complexation towards NOM in the case of Cu and Al, while Mn remained mainly as free ions.
- From an environmental fate and risk assessment perspective, the results imply that Cu, Mn, and Al NPs will dissolve relatively rapidly in the presence of DHBA and humic acid, and any NP-specific risks (*e*.*g*. increased toxicity) are therefore limited to the vicinity of their dispersion source. With no or very little NOM present, the release is much slower, but on the same time there is less stabilization of the NPs, hence limiting their mobility.
- The study elucidates that the fate of metal NPs of different reactivity and non-metallic NPs is dissimilar due to differences in surface reactivity, stability, mobility and dissolution characteristics, aspects that need to be considered in NP fate modeling and risk assessments.
